# Academic and non-academic predictors of academic performance in medical school: an exploratory cohort study

**DOI:** 10.1186/s12909-022-03436-1

**Published:** 2022-05-13

**Authors:** Marija Franka Žuljević, Ivan Buljan

**Affiliations:** 1 Department of Medical Humanities, School of Medicine, University of Split, Šoltanska 2, 21000 Split, Croatia; 2 Department of Research in Biomedicine and Health, School of Medicine, University of Split, Split, Croatia

**Keywords:** Medical students, Predictors, Academic performance, Medical education

## Abstract

**Background:**

Medical schools should also evaluate applicants’ non-academic characteristics in the search for successful students and future physicians, but ideal non-academic criteria have not yet been found. We followed two successive generations of medical students at the University of Split School of Medicine (USSM) to assess both academic and non-academic constructs as predictors of academic performance, defined as medical school grade point average (GPA). We also interviewed some of the participants to gain additional insight for future studies.

**Methods:**

We measured study GPA in first and last year, as well as attitudes towards science, motivation, emotional intelligence, self-esteem, and perceived personal incompetence in first year. We also obtained their scores on existing medical school enrollment criteria, the State Graduation Exam (SGE) and high-school GPA. Regression models were constructed for predictors of GPA in the last year of medical school. Four structured pilot interviews were conducted to explore participants’ perceptions of necessary traits for medical school and later practice.

**Results:**

Regression analysis showed that only SGE predicted final academic performance in medical school (β=0.35, 95% confidence interval (CI)=0.06-0.64), while none of the non-academic constructs we assessed predicted this outcome of education. The two generations did not significantly differ in any variable except that intrinsic motivation was higher in the generation that enrolled in 2011 (OR=1.47, 95%CI=1.12-1.93, *P*=0.005).

**Discussion:**

None of the non-academic constructs predicted academic performance in medical school. Their use as selection criteria may not be warranted as they could impact the academic quality of enrolling medical students.

**Supplementary Information:**

The online version contains supplementary material available at 10.1186/s12909-022-03436-1.

## Background

The value of an admission criterion for medical school is its ability to predict the applicant’s performance during undergraduate medical training, as well as after graduation [1, 2]. However, it is very difficult to reach a consensus on the most desirable personal qualities of medical school applicants, and it is equally difficult to reliably measure them [3]. Most current established methods of selecting suitable students are cognitive and knowledge-based assessments [4]. Most of these, such as the high-school grade point average (GPA) and medical school aptitude tests, have been established to be good predictors of students’ academic performance [5, 6]. However, a systematic review showed that academic scores account for only 23% of the variance of progress measures at medical school [6]. The discriminatory power of academic tests may be decreasing, as more students now get top grades [7]. Some argue that academic performance may be necessary for creating a competent clinician later on, but that it alone is not sufficient [7]. The current opinion is that medical schools should also evaluate applicants’ non-academic characteristics [8–11]. Additionally, in light of the COVID-19 pandemic, many Ivy League institutions in the US have pledged “test-optional” policies for standardized admission testing, while some of them have also argued that these tests discriminate against certain applicants and should be eliminated [12–14].

There are mixed results on whether non-academic constructs are useful as admission tools for medical school [4]. Only a few non-academic assessment methods, like the multiple mini interview [15], have successfully been implemented into practice. Self-reported personality factors show a significant relationship with medical school academic performance [16], but their relationship is complex and nonlinear, and the evidence on their use is inconclusive [7]. Their use could potentially reduce the diversity of applicants and their long-term predictive validity is insufficiently explored [7]. Emotional intelligence (EI) assessments could be a valuable tool in future selection [7], although EI has only been explored in mostly preliminary studies [17–19]. A meta-analysis found that psychological factors such as motivation and self-esteem, among others, show some potential in predicting the academic GPA of university students in general [20]. Motivation may also positively affect academic performance in medical school [21, 22]. “Non-cognitive” traits assessed in the UK Clinical Aptitude Test were evaluated and a weak correlation of self-esteem and academic performance in medical school was found [23], but self-esteem was linked with a higher clinical competence [24].

Ideal non-academic criteria for entry into medical school have not yet been found [25]. Well-structured cohort studies exploring non-academic constructs are still lacking [7]. The questions raised on the validity of non-academic tests and the fairness of their use in a high-stakes evaluation have also not been answered [26]. Harris et al have emphasized the fact that non-academic tests have not shown good validity in predicting academic performance in comparison with traditional academic tests, and that their implementation is not fully warranted by evidence [27]. Students could be coached to give desirable answers to non-academic admission tests, making this a potential barrier to implementing such assessment methods [1]. Suggested use of a social desirability scale to detect false responses did not fully address the issue [28]. With the lack of the evidence about the predictive validity of non-academic measures, which would bring the incremental value to existing measures, more research is warranted.

To address the existing gaps in knowledge, we aimed to explore the ability of existing academic assessment methods and non-academic constructs to predict final-year academic performance, defined as study grade point average GPA, in two successive generations of medical students at the University of Split School of Medicine (USSM). As this was an exploratory study, we decided to focus on non-academic constructs that were previously inconclusive as academic predictors: EI, motivation, and self-esteem; as well as two constructs previously unexplored in medical students: attitudes towards science and perceived personal incompetence. To measure them, we used questionnaires that were previously validated in our setting. To gain additional insight about the student perceptions about medical studies program and their motivations, we also conducted interviews with a smaller number of study participants.

## Methods

### Study design and setting

This study was an exploratory prospective cohort study and was performed at the USSM, a medical school in Split, Croatia. Medical schools in Croatia, as in most European countries, are integrated undergraduate and graduate studies resulting in a degree of medical doctor (MD).

#### USSM enrolment scheme

Students need to pass the State Graduation Exam (SGE) at the end of their high school education to graduate and qualify for enrolment in the USSM medical school program. Information on the SGE structure and scoring, as well as some changes in enrolment requirements from 2010/2011 to 2011/2012 are described in Additional file 1.

#### Structure of the USSM program

The USSM program consists of six academic years of study: the first three include pre-clinical subjects, and the last three are clinical and involve contact with patients [29]. Academic performance is measured by grades on a scale ranging from 2 – pass to 5 – outstanding. The study grade point average (GPA) on a scale 2.0-5.0 is therefore used and can be calculated for each study year separately, as well at the total study years together. We chose this GPA as the main outcome measure of participants’ academic performance, especially since study GPA is important criterion for receiving scholarships and accommodation within student dorms in the Croatian education system. Likewise, Croatian medical students’ GPA is important when applying to clinical residencies and is one of the main parts of their admission scoring system.

### Participants

Participants were medical students at the USSM from two generations that enrolled in the program during the academic years 2010/2011 and 2011/2012. These two generations made up two different cohorts that were followed during this study. We excluded students repeating the first year, as well as those partially repeating first year.

#### Cohort 2010/2011

A total of 86 students were enrolled into the 2010/2011 generation. They were required to complete the SGE before enrolment, but to qualify for enrolment, they were also required to pass obligatory exams in Biology, Chemistry, and Physics as a part of the SGE.

#### Cohort 2011/2012

A total of 82 students enrolled into the 2011/2012 generation. They also had to complete the SGE before enrolment, but had no obligatory exams required apart from the basic exams in the SGE.

### Questionnaire

At both data collection points, participants were asked to provide their current GPA (on a scale 2.0-5.0), and this was considered the main outcome measure of their academic performance. They were also asked if they were partially enrolled in or repeating the academic year during which they were surveyed. At baseline (first study year), we asked them to provide basic demographic information (age at enrolment, gender), their score on the SGE, and their GPA at the end of high-school education (scale 2.0-5.0). They were then asked to fill out the following questionnaires: Attitudes Towards Science Scale [30], Work Preference Inventory [31], Emotional Skills and Competence Questionnaire (ESCQ-45) [32], Rosenberg Self-esteem Scale [33], Perceived Personal Incompetence Scale [34]. Detailed descriptions of the questionnaires are given in Additional file 2.

### Data collection

Data were collected (by IB) at two points during the participants’ medical education using a paper-based survey. Additional information on the survey setting, data collection time points, and pairing of participant responses is provided in Additional file 1.

#### Missing data

A participant’s answers were included in the data analysis if we collected their data for both measurements. If participants were repeating the first year, we concluded they would be excluded from the final analysis, as we only wanted to follow the original cohorts, i.e. students who enrolled for the first time. Participants were also excluded from the final analysis if they had any questionnaires missing, i.e. began completing the survey, and then gave up midway or at the beginning. We calculated the average score for each item and replaced each missing response with the average item score. This way, the overall result was not affected, and we did not need to exclude participants due to missing items.

### Study size

The sample size calculation was performed using an online sample size calculator [35]. Based on previous research results on academic performance [20], where the percentage of variance explained was R^2^=0.28 (f^2^=0.50), we calculated (β=0.8, α=0.05) that we will need, at minimum, 45 participants to perform a regression analysis with eleven predictors.

### Data analysis

All statistical analyses were performed using JASP software v. 0.13.1.0 (JASP Team, 2018, Amsterdam, Netherlands) and MedCalc software v. 19.5.3 (MedCalc Software Ltd, 2020).

#### Demographic data and response rate

Gender and response rate was expressed for both generations as frequencies and percentages. Response rate was calculated for each generation and compared by a chi-square test (χ^2^).

#### Descriptive statistics and comparison of cohorts

Results for all collected variables were calculated separately for the 2010/2011and 2011/2012 generations and expressed using a median with 95% confidence interval (CI) after determining data distribution for each variable by using the Shapiro-Wilk test. The Mann-Whitney independent samples test was then used to compare the two generations for each variable. A Bonferroni correction for multiple measurements was performed (0.05/12) and determined the significance cut-off for the comparisons to be *P*=0.004. Significant variables were entered into a stepwise logistic regression to confirm any differences, with results expressed as odds ratio (OR), a corresponding 95%CI, and P-value.

#### GPA prediction

As first-year and final-year GPA are linked variables, their correlation was estimated using Pearson’s r. We used stepwise linear regression to assess predictors of final-year GPA. Results were expressed as standardized regression coefficients (β) with 95%CI, along with the coefficient of determination (R^2^) for significant predictors. We calculated the 95%CI for β using the following equation [36]: 95%CI=β±1.96∙(β/t_value_).

#### Baseline participant characteristics and dropout

We used the Mann-Whitney independent samples test to compare all variable scores between participants at baseline that were included in the study, and those that were not included in the study (i.e. filled out the survey only in first year). Results were expressed as P-values with a significance cut-off of 0.05. For additional confirmation, we entered significant results into a logistic regression model with the results expressed with an odds ratio (OR) and 95%CI.

### Ancillary pilot interviews

One of the authors (IB), a teacher at the USSM, conducted four structured interviews in June 2017 with sixth-year USSM medical students, who were also participants in the quantitative part of the study and volunteered to additionally do the interviews. The interviews’ main purpose was to provide a brief insight into participants’ personal reflections and observations to help inform and refine which constructs should be explored in future studies. Students were asked about their experience, attitudes and perceptions on their medical studies, as well as what a good physician is, in their personal opinion (questions shown in Additional file 3). The participants had no previous relationship with the interviewer. A more detailed description of the interview methodology is available in Additional file 1. The COnsolidated criteria for REporting Qualitative research (COREQ) checklist [37] was used to guide our reporting (available in Additional file 4).

## Results

### Demographic data and response rate

**Fig. 1:**
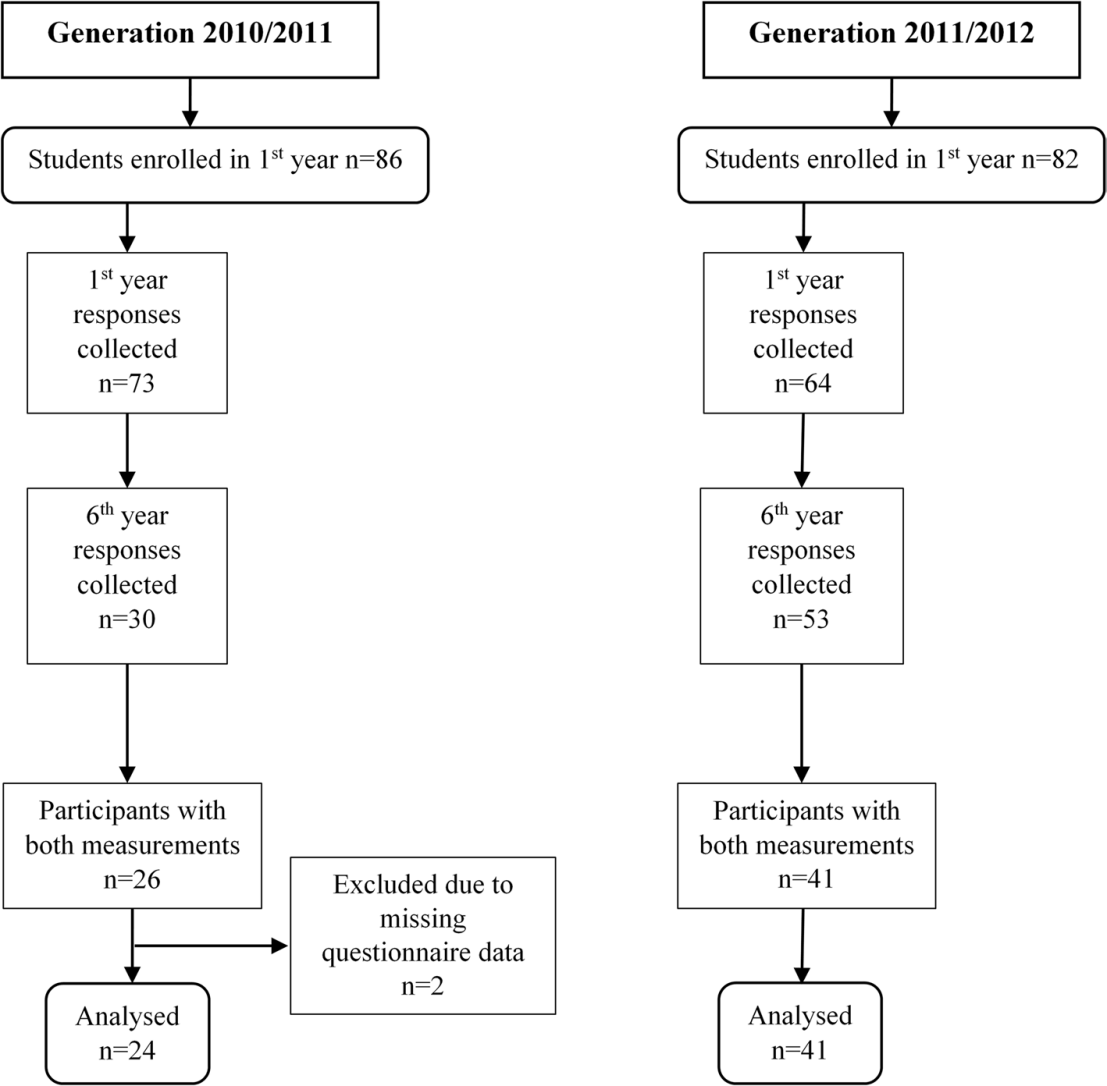
Flowchart showing the number of participants at each stage in the study

Overall, 65 students were included in the final analysis. We constructed a flowchart to show the numbers of individuals involved at each stage of study (Fig. 1).

Generation 2010/2011 had 24 participants, with a female majority (*n*=18, 75%) and a response rate of 27.9% (24/86). The 2011/2012 generation had 41 participants, with a female majority as well (*n*=31, 75.6%). The response rate was 50% (41/82) and was higher than for generation 2010/2011 (χ^2^=8.63, *P*=0.003).

### Descriptive statistics and comparison of cohorts

The two generations did not significantly differ in any variable except their motivation, where generation 2011/2012 had higher intrinsic motivation (*P*<0.0001) and extrinsic motivation (*P*=0.0002) scores (Table 1). A stepwise logistic regression model only confirmed intrinsic motivation to be significantly different (OR=1.47, 95%CI=1.12-1.93, *P*=0.005), while extrinsic motivation and other variables were not.

**Table 1:** Descriptive statistics and comparison of scores for generations 2010/2011 and 2011/2012

Variable (scale range)	Median (95%CI)		P*
	Generation 2010/2011 (*N*=26)	Generation 2011/2012 (*N*=41)	
High-school GPA (2.0-5.0)	4.8 (4.7-5.0)	4.9 (4.8-4.9)	0.505
SGE score (maximum score 1000.0)	762.0 (693.9-908.0)^a^	820.0 (800.0-847.1)^b^	0.321
First year GPA (2.0-5.0)	4.0 (3.9-4.3)^c^	4.0 (4.0-4.2)^d^	0.675
Sixth year GPA (2.0-5.0)	4.1 (4.0-4.2)	4.0 (3.9-4.1)	0.534
Attitudes towards science			
Perceived value for humanity (16.0-80.0)	59.0 (52.0-65.7)	56.5 (54.3-60.7)	0.551
Perceived value of scientific methodology (12.0-60.0)	44.0 (40.8-49.5)	46.0 (43.0-48.0)	0.568
Perceived value for medicine (17.0-60.0)	61.0 (58.3-66.0)	62.0 (59.0-65.0)	0.721
Work Preference Inventory			
Intrinsic motivation (15.0-75.0)	47.5 (45.8-51.0)	62.0 (59.0-64.0)	**<0.001**
Extrinsic motivation (15.0-75.0)	42.0 (38.8-43.3)	49.0 (46.9-51.5)	**<0.001**
ESCQ-45 (45.0-225.0)	169.5 (160.5-176.3)	172.0 (165.0-179.5)	0.373
Rosenberg self-esteem scale (10.0-50.0)	41.5 (36.0-44.3)	44.0 (41.0-45.0)	0.223
Perceived incompetence (10.0-50.0)	21.0 (14.8-25.3)	16.0 (14.8-19.0)	0.065

^*^Mann-Whitney independent samples test. A Bonferroni correction for multiple measurements was performed (0.05/12) and determined the cut-off as *P*=0.004

*GPA* Grade Point Average, *ESCQ-45* Emotional Skills and Competence Questionnaire

^a^20 participants did not provide their SGE score

^b^8 participants did not provide their SGE score

^c^One participant did not provide their first-year GPA score

^d^6 participants did not provide their first-year GPA score

### GPA prediction

We estimated a very high correlation between first-year and sixth-year GPA (*r*=0.745, *P*>0.001), confirming the linked nature of the two variables. Based on this, we chose not to include first-year GPA in our stepwise linear regression model [38]. However, we included all other academic variables (high-school GPA, SGE score) and all surveyed non-academic variables (attitudes on science, motivation, EI, self-esteem, perceived incompetence). Only the SGE score was a significant predictor of final academic performance in medical school (β=0.35, 95%CI=0.06-0.64), explaining 12.3% of variance of sixth-year GPA scores.

#### Baseline participant characteristics and dropout

Participants that completed the study had a higher intrinsic motivation (*P*=0.009) than students who only filled out the survey in first year and were then lost to follow-up. There was no significant difference in any other variable (data not shown). Results of a logistic regression model also showed that intrinsic motivation significantly predicted whether a student completed the study, although the effect size was small (OR=1.05, 95%CI=1.01-1.09).

### Ancillary pilot interviews

Four participants were approached for the interviews, and all of them agreed to participate. The final sample of interviewed students consisted of four medical students (female sixth year students, age range 23-24 years, GPA range 3.70-4.96).

We present the most relevant findings below. Participant quotes, along with the full division into themes and sub-themes, are available in an additional file (Table 1.3 in Additional file 3).

#### Perception of a “good physician”

When participants were asked on how they perceive a good physician, both technical knowledge and personal characteristics and traits related to professionalism were perceived as necessary. Three participants placed a special emphasis on empathy.

#### Medical studies’ role in education

Three participants elaborated that medical studies cannot teach non-academic skills such as empathy and that these values are already pre-existing in a person. Some participants stated that their ability to empathize actually decreased over the course of their medical education.

#### Selection of students for medical school

Perception of academic-based selection methods such as the SGE or entrance exams was that they are good methods of selecting students with a willingness to learn and good technical knowledge, but not for favorable personal characteristics. Some participants observed that there are other medical students who are poorly fit to be future physicians, and that some experience mental disorders during the course of their studies in a way that also makes them less fit for this role.

#### Predictors of academic success in medical school

Perseverance was perceived by all four participants to be the main predictor of academic success for medical students, with some participants placing exclusive emphasis on perseverance in terms of studying and fulfilling obligations.

## Discussion

Our results show that only the pre-admission knowledge test (SGE), predicted the overall academic performance of medical students. EI, motivation, and self-esteem, attitudes towards science, and perceived personal incompetence showed no ability to predict GPA in medical school, in the sample of participants who completed six years of medical school. However, participants that completed the study, i.e. those who completed their medical education within 6 years, had a higher intrinsic motivation than those lost to follow-up, indicating that intrinsic motivation was predictive for medical study program attrition, however, with only a small, non-practical, effect.

Even though previous research has emphasized the need to use students’ non-academic characteristics when selecting medical students [8–11], our study did not find them to be significant predictors of academic performance. Our results support the preliminary findings on EI [17] and self-esteem [23] which previously showed no significant association with academic performance. However, our findings on motivation are different from past studies which found merit in its predictive ability for academic performance [21, 22]. There are no studies on the association of attitudes towards science and perceived personal incompetence thus far, and our findings can be considered preliminary in this context. Our study is also one of the first to assess the predictive ability of the SGE, along with Ravlić-Gulan et al. who initially described its association with academic performance [39]. However, they found that a change in SGE enrolment criteria between study generations impacted academic success, which was not observed in our study. Our findings on the SGE are similar to what is known about the SAT tests in the US, which are also taken at the end of high-school education and seem to have some predictive ability for early academic performance in medical school [40, 41]. However, we found that the SGE also predicts academic performance in later clinical years.

Newly implemented admissions systems with non-academic criteria could select students with low academic credentials and result in them causing problems for the faculty [3]. Such situations could thus cause additional resistance to implementing any new admissions system [3]. We reaffirm that a non-academic construct still needs to be able to predict academic performance. As other researchers reported, the implementation for the non-academic constructs that we assessed may also not be warranted by evidence [9]. Their potential importance cannot be excluded, but there is a risk of a decrease of academic quality of students if they are included as admission criteria, especially in settings such as in our study. Academic outcomes are deeply embedded within the curriculum in the Croatian setting and are important to the advancement in a student’s medical career. It also has a long-standing tradition of using academic admission criteria. A 30-year retrospective study at the USSM found that high-school GPA and entrance exam scores (used before the SGE was introduced) predicted medical school GPA [42]. For our setting, these findings imply that a classical entrance exam is not necessary for medical school with a standardized national test like the SGE in place. Overall, even though we agree that including non-academic admission criteria would be more fair, we highlight that the problem of coaching for socially desirable answers remains a significant barrier to their implementation as admission tests in practice, even if their predictive ability is observed [1, 28].

A strength of our study is its prospective cohort study design, as other studies on this subject are mostly cross-sectional [6]. Even though we had a smaller sample size, any existing predictive effects should be evident in a smaller sample. Our observed predictive ability of academic constructs is therefore more likely to represent a true effect. The two surveyed generations differed only in motivation scores, something we considered as adequate rationale for analyzing them as a single sample. Still, our study has certain methodological limitations. Even though our overall response rate was satisfactory, we still experienced significant participant dropout [43]. This is the reason we conducted an additional analysis of attrition bias. Due to the anonymous nature of our surveys, we cannot be sure about the reason the students did not make it to the final year of study. Likewise, the length of our survey may have caused fatigue for participants, so we provided candy bars as participation incentives [44]. Our choice GPA is also not fully ideal, as it is a rough outcome measure. However, it was the best and most objective measure that we could select within our setting and one that is also very relevant to our participants’ future education and training. Alternative assessment methods would depend on teacher assessment, could be prone to bias, and would disrupt the anonymous nature of the study. Self-reported GPA is also a measure that may be subject to recall bias. However, due to the high emphasis on GPA in our setting, we believe that the potential for recall bias is low, as the students are very well aware of their GPA, especially in the first (high-school students come prepared for GPA importance as it is equally important in high school and for the enrolment at a university) and last years of medical school (they are monitoring their GPA as it impacts the application for all career options immediately after obtaining their medical doctor degree). Therefore, we assert that the potential for recall bias is no more than is usual for survey studies in general.

Overall, as the constructs assessed in this study may not be adequate candidates as admission criteria, our findings may serve as a guide to better focus future research efforts on constructs that can hold up to more rigorous assessment in their predictive ability for a variety of outcomes. Future studies could consider also looking at outcomes such as the OSCE, which are often formally assessed as part of a medical school curriculum [45, 46]. We encourage the search for other non-academic constructs that can predict academic success, especially using a multi-site longitudinal study design. Based on the pilot interviews we conducted, we found that participants highlighted empathy as an important trait for a future physician and perseverance for fulfilling academic obligations in medical school. Interviewees also reaffirmed the necessity of finding adequate non-academic constructs to screen students. Although the preliminary findings on the predictive ability of empathy for academic performance are not particularly encouraging [17], future studies on its association with important outcomes of education could be insightful. Perseverance i.e. grit could also be explored further [47, 48], especially its association with study success is already known in a non-medical setting [49].

## Conclusion

Our exploratory analysis of two successive cohorts of medical students at the University of Split School of Medicine showed that the State Graduation Exam score, a standardized knowledge test, predicted students’ academic performance in medical school, i.e. the final GPA at the end of the medical studies. We assessed several constructs that have either been unexplored or previously had preliminary findings only: EI, motivation, self-esteem, attitudes towards science, and perceived personal incompetence, and found that none of them predicted overall academic performance in medical school. Based on their inability to predict an important outcome of education, their use as selection criteria for medical school may not be warranted at present, as it could impact the academic quality of enrolling students. Further research efforts should be focused on assessing different non-academic constructs in longitudinal studies, such as perseverance and empathy.

## Supplementary Information

**Additional file 1.**

**Additional file 2.**

**Additional file 3.**

**Additional file 4.**

## Data Availability

The dataset supporting the conclusions of this article is available in the Open Science Framework repository, http://doi.org/10.17605/OSF.IO/7RQY3.

## Abbreviations

Β: Standardized regression coefficient
95%CI: 95% confidence interval
EI: Emotional intelligence
ESCQ-45: Emotional Skills and Competence Questionnaire
GPA: Grade point average
OR: Odds ratio
OSCE: Objective Structured Clinical Examination
SGE: State Graduation Exam
USSM: University of Split School of Medicine
