# Supplementary material for: Academic and non-academic predictors of academic performance in medical school: an exploratory cohort study
Source: BMC Med Educ. 2022 May 13;22:366. doi: 10.1186/s12909-022-03436-1 (PMC9098375; doi:10.1186/s12909-022-03436-1)
Supplement: Supplementary file 1 — Additional file 1. [file 12909_2022_3436_MOESM1_ESM.pdf]

Additional file 1: Supplementary information on the study methodology

University of Split School of Medicine enrolment scheme

The Croatian State Graduation Exam (SGE) has three obligatory subjects which students must pass to graduate from high school: Croatian language, Mathematics, and an additional foreign language. Students can obtain a maximum of 1000 points on the Exam, but each institution of higher learning standardizes this to their own scoring criteria, leaving the total maximum score unchanged. Enrolment requirements for our study setting, the University of Split School of Medicine, are shown in **Table 1.1**. In the academic year 2010/2011 the USSM required applicants to also pass obligatory examinations in Biology, Chemistry and Physics to qualify for enrolment. However, this enrolment requirement was removed in the academic year 2011/2012.

Table 1.1. Enrolment requirements at the University of Split School of Medicine for the academic years 2010/2011 and 2011/2012.

Academic year	Prerequisite conditions	High-school grade evaluation (% total score)	Mandatory SGE exams (% total score)	Elective SGE Exams (% total score)	Other (% total score)	Total score at enrolment
2010/2011	Completion of SGE; attendance of at least two years of Biology, Physics, Chemistry and Latin language for at least two years of secondary school education	25%: GPA on a scale 2.00-5.00	33%: English language (10%), Croatian language (10%), Mathematics (13%)	30%: Biology (10%), Physics (10%), Chemistry (10%)	2%: awards at state competitions in high-school	1000
2011/2012	Completion of SGE	25%: GPA on a scale 2.00-5.00	75%: English language (25%), Croatian language (25%), Mathematics (25%)	-	-	1000

Abbreviations: SGE=State Graduation Exam, GPA=Grade Point Average

Data collection

The 2010/2011 generation was assessed during their first study year in June 2011 and their sixth study year in February 2016. The 2011/2012 generation was assessed during their first study year in June 2012 and their sixth study year in February 2017. Survey completion took an average of 40 min. Students received a candy bar as an incentive for participation in the survey.

At the beginning of the survey, we asked the students to generate a five-element unique identifier code to pair their responses at all three data collection points, using methodology previously described in similar studies [1, 2]. The code consisted of the first letter of the participants' name, first letter of their

mothers' name, first letter of their fathers' name, last two digits of their birth year, and first letter of the place they were born.

Ancillary pilot interviews

Four participants were recruited through purposive sampling and approached face-to-face. The interviewer (IB) has completed a PhD and is a trained psychologist who also specializes in cognitive behavioral therapy (CBT). He has extensive previous experience with qualitative research [3, 4, 5]. The purpose of the interviews and the study was explained to participants and they gave verbal informed consent to the interviewer and agreed to be recorded during the interview. They were also informed of the interviewer's professional credentials. The interviews were conducted at the USSM, and no one was present except for the interviewer and participant for each interview. The participants were not previously taught by the interviewer, as he was only recently employed at the USSM and had not held any classes for the cohorts involved in the study. The interviewer was also close in age to the students, which may have had a positive impact on their ability to elaborate their opinions freely. Participants first filled out basic demographic information (age, year of study, GPA). The interview was conducted according to pre-defined questions, and the participants provided their answers without additional prompts. The interview questions were not piloted before the interviews, as this already was a pilot part of the study. Duration of the interviews was between 10 and 12 minutes. No repeat interviews were carried out and the interviewer did not make field notes. Data saturation was not considered applicable in this case. One author (MFŽ) coded the transcripts and they were not returned to participants for comment. The interviews were recorded and another author (MFŽ) later transcribed the interviews and translated them into English, and the interviewer (IB) assessed the translation to confirm the accuracy. One author (MFŽ) coded the transcripts using Microsoft Excel, using a phenomenological and pragmatist approach. Themes were identified in advance according to the planned interview questions. Sub-themes were coded inductively. Participants did not provide feedback on the findings.

References

1. Hren D, Marusic M, Marusic A. Regression of moral reasoning during medical education: combined design study to evaluate the effect of clinical study years. PloS One. 2011;6(3):e17406. doi: 10.1371/journal.pone.0017406

2. Bokan I, Buljan I, Marusic M, Malicki M, Marusic A. Predictors of academic success and aspirations in secondary nursing education: A cross-sectional study in Croatia. Nurse Educ Today. 2020;88:104370. doi: 10.1016/j.nedt.2020.104370

3. Tomić V, Buljan I, Marušić A. Perspectives of key stakeholders on essential virtues for good scientific practice in research areas. Account Res. 2022;29(2):77-108. doi: 10.1080/08989621.2021.1900739

4. Buljan I, Barać L, Marušić A. How researchers perceive research misconduct in biomedicine and how they would prevent it: A qualitative study in a small scientific community. Account Res. 2018;25(4):220-238. doi: 10.1080/08989621.2018.1463162

5. Buljan I, Tokalić R, Roguljić M, Zakarija-Grković I, Vrdoljak D, Milić P, Puljak L, Marušić A. Comparison of blogshots with plain language summaries of Cochrane systematic reviews: a qualitative study and randomized trial. Trials. 2020;21(1):426. doi: 10.1186/s13063-020-04360-9
