# Supplementary material for: Academic and non-academic predictors of academic performance in medical school: an exploratory cohort study
Source: BMC Med Educ. 2022 May 13;22:366. doi: 10.1186/s12909-022-03436-1 (PMC9098375; doi:10.1186/s12909-022-03436-1)
Supplement: Supplementary file 2 — Additional file 2. [file 12909_2022_3436_MOESM2_ESM.pdf]

**Additional file 2: Detailed descriptions of all questionnaires included in the survey**

Students filled out the following questionnaires:

1. Attitudes towards science

Attitudes towards science were measured by the 45-item Attitudes Towards Science Scale (1), which has 3 sub-scales: Value of science to humanity, Value of scientific methodology, and Value of science to medicine. Each sub-scale was treated as a separate variable. The assessment was based on a five-point Likert-type scale ranging from 1 – Completely disagree to 5 – Completely agree (maximum score 225). Higher scores indicated more positive attitudes towards science. Cronbach's alpha was $\alpha$=0.91 (95% confidence interval (CI)=0.89-0.93).

Value of science to humanity was assessed by 16 items (Nos. 1, 4, 7, 10, 13, 16, 19, 22, 25, 28, 31, 34, 37, 39, 41 and 43, score range 16-80). Items No. 1, 7, 13, 16, 19, 28, 31, 34, 37, 39 and 43 were reversely scored.

Value of scientific methodology was assessed by 12 items (Nos. 2, 5, 8, 11, 14, 17, 20, 23, 26, 29, 32 and 35, score range 12-60). Items No. 8, 14, 23 and 26 were reversely scored.

Value of science to medicine was assessed by 17 items (Nos. 3, 6, 9, 12, 15, 18, 21, 24, 27, 30, 33, 36, 38, 40, 44 and 45, score range 17-60). Items No. 12, 18, 27, 30 and 36 were reversely scored.

2. Motivation

Motivation was assessed by the 30-item Work Preference Inventory (2), which measures two aspects of motivation: extrinsic (EM) and intrinsic (IM). We treated each motivation type as a separate variable. The assessment was based on a five-point Likert-type scale ranging from 1 – Completely disagree to 5 – Completely agree (maximum score of 150). Higher scores indicated an individual having a higher self-perceived motivation level.

Extrinsic motivation was assessed by 15 items (Nos. 1, 2, 4, 6, 10, 12, 15, 16, 18, 19, 21, 22, 24, 25, and 29, score range 15-75). Items No. 1, 16, and 22 were reversely scored; Cronbach's alpha was $\alpha$=0.80 (95% CI=0.73-0.84).

Intrinsic motivation was assessed by 15 items (Nos. 3, 5, 7, 8, 9, 11, 13, 14, 17, 20, 23, 26, 27, 28, and 30, score range 15-75). Items No. 9 and 14 were reversely scored; Cronbach's alpha was α=0.88 (95% CI=0.85-0.91).

3. Emotional skills and competence

Emotional intelligence was assessed using the 45-item Emotional Skills and Competence Questionnaire (ESCQ-45) (3), which was developed using the model of Emotional Intelligence by Mayer and Salovey (4). ESCQ-45 has three sub-scales: Perceiving and understanding emotions, Expressing and labelling emotions, and Managing and regulating emotions. The assessment was based on a five-point Likert-type scale ranging from 1 – Never to 5 – Always (maximum score of 225). Higher scores indicated a higher overall EI. Cronbach's alpha was α=0.90 (95% CI=0.87-0.92).

4. Self-esteem

Self-esteem was assessed using the 10-item Rosenberg self-esteem scale (5), where the assessment was based on a five-point Likert-type scale ranging from 1 – Completely disagree to 5 – Completely agree (maximum score 50). Items No. 2, 3, 4, 6, and 9 were reversely scored. Higher scores indicated a higher sense of self-worth. Cronbach's alpha was α=0.84 (95% CI=0.79-0.88).

5. Perceived personal incompetence

Perceived personal incompetence was assessed with a 10-item scale (6), where the assessment was based on a five-point Likert-type scale ranging from 1 – Completely disagree to 5 – Completely Agree (maximum score 50). Higher scores indicated that a participant perceived themselves to be less competent, while lower scores meant they self-reported a higher sense of personal competence. Cronbach's alpha was α=0.88 (95% CI=0.85-0.91).

**References**

1. Hren D, Lukic IK, Marusic A, Vodopivec I, Vujaklija A, Hrabak M, et al. Teaching research methodology in medical schools: students' attitudes towards and knowledge about science. Med Educ. 2004;38(1):81-86. doi: 10.1111/j.1365-2923.2004.01735.x

2. Amabile TM, Hill KG, Hennessey BA, Tighe EM. The Work Preference Inventory: assessing intrinsic and extrinsic motivational orientations. J Pers Soc Psychol. 1994;66(5):950-967. doi: 10.1037//0022-3514.66.5.950

3. Takšić V, Mohorić T, Duran M. Emotional skills and competence questionnaire (ESCQ) as a self-report measure of emotional intelligence. Horizons of Psychology. 2009;18(3):7-21.

4. Mayer JD, Salovey P. What is emotional intelligence: implications for educators. In: Salovey P, Sluyter D, eds. Emotional Development and Emotional Intelligence: Educational Implications. New York, NY: Harper Collins; 1997:3-24.

5. Rosenberg M. Society and the adolescent self-image. Princeton, NJ: Princeton University Press; 1965.

6. Bezinović P. Percepcija osobne kompetentnosti kao dimenzija samopoimanja. [doctoral dissertation]. Zagreb, Croatia: Filozofski fakultet Sveučilišta u Zagrebu; 1988.
