# Supplementary material for: Academic and non-academic predictors of academic performance in medical school: an exploratory cohort study
Source: BMC Med Educ. 2022 May 13;22:366. doi: 10.1186/s12909-022-03436-1 (PMC9098375; doi:10.1186/s12909-022-03436-1)
Supplement: Supplementary file 3 — Additional file 3. [file 12909_2022_3436_MOESM3_ESM.pdf]

Additional file 3: Results of the qualitative part of the study

Questions asked in the interview:

1. What makes a good physician?
2. What role does medical school have in creating a good physician?
3. What do you think about the current selection of students through the State Graduation Exam? Is there a better method?
4. What is, in your opinion, the main predictor that students have a good GPA?

Table 1.3. Themes and quotations from interviews with medical students.

**Theme and characteristic**	**Quote**
**Perception of a "good physician"**	
Technical knowledge	I: What makes a good physician?<br>P2: To have expertise. (…) But again, someone can be an expert, but if they are a bad person, they are not a good physician. But if someone is good, but knows nothing, again…<br>P4: Punctuality, responsibility, knowledge. Of course, knowledge, this goes without saying.
Personal characteristics and traits related to professionalism	I: What makes a good physician?<br>P1: Taking the job seriously, which I think most don't. (…) And empathy – which should somehow be [learned]…<br>P2: Perseverance is important. First, one needs to love this job. And to be a good person, this comes first.<br>P3: What makes a good physician is, besides knowledge which should certainly be present, is a sort of empathy, that you accept the fact that you work in a hospital, that the people who come to see you are mostly sick people with their own pre-existing problems…<br>P4: …and this empathy, which not everyone possesses.
**Medical studies' role in education**	
Insignificant role in creating good physicians	I: What role does medical school have in creating a good physician?<br>P1: It should teach you [how to become one] – but again, it's up to how much a person wants to learn.<br>P2: Maybe relating to knowledge, yes. The rest, not really, I think.<br>P3: In my opinion, not a very big one... Regarding the knowledge we gain, I could have sat at home for six years, book in hand, studying. There were very little seminars and lectures where we actually learned something new. We lost a lot of time here. About these other things, obligations and so on, that it teaches you a sense of responsibility – I think it hasn't done much here.<br>P4: It could be bigger. It does not currently have as big a role as it should have…<br>P4: In my opinion, [a student] can turn out good regardless of, say, medical school.

Not good at teaching non-academic skills	P1: …unfortunately, you can't teach [empathy] in medical school, it's something one's parents were supposed to teach, but they didn't.<br><br>P4: I think [empathy] is something that is brought from home. You simply learn about it… either you're that way, or you aren't. No one can teach you in medical school.<br><br>P3: ...these people didn't learn anything about responsibility and obligations. While some other people already had it in themselves only sharpened these skills…
May decrease empathy	P1: I am not more or less empathetic when I was [before my studies]. Actually, maybe I am less – meaning I've seen some ugly situations and that it will now be easier to deal with something like death.<br><br>P4: It seems to me that medical school decreases this empathy. The more people you see, it's somehow easier to move on from what you've seen, or when you meet a person with a similar disease. Or –it's easier each time. It's not that you don't have empathy, but you just get used to it. You become numb.
**Selection of students for medical school**	
Some medical students are poorly selected	P1: For example, my generation has some people… I'm not sure if it's not nice to say, but I think that they have a diagnosis. So… they shouldn't be here where they are (laughs).<br><br>P1: So I think there are many people here who have quite strayed from their path.<br><br>P4: In my opinion, there are bad people who were not that way in the beginning. Simply, this lifestyle where you have to study all the time… it influences it. It is a matter of time until you develop a psychological disorder for which you maybe had a predisposition before.
Current methods are good for selection of students who can develop good technical skills in medicine	I: What do you think about the current selection of students through the SGE?<br><br>P1: The SGE shows whether you are ready or not to study in high school. Entrance exams are nothing but the SGE, again, there is no difference, in my opinion. The only difference is that, when we were enrolling, there was no biology, I think it's different now, or not… but that's OK, to include another subject that has something to do with [medicine], not only, say, mathematics, which has nothing to do with what kind of physician someone will be one day…<br><br>P2: I am not so sure. It might be better to bring back the entrance exam.<br><br>P2: So… as far as I know now, now they included these elective subjects as well. This is OK. When we were enrolling, this was all 25% of the score. So, GPA, Croatian, mathematics, English. So we got this, let's say, rather, let's quote the people from the University, that this was a "terrible generation". So that was bad, and now... I guess this is OK, although I don't see that the generations after us are any better.<br><br>P3: I don't know what to say… In my opinion, this entrance exam that everyone wants to include is not that crucial either, because if someone is made for [medical school] and they can do it, they will also enroll. Anyone can enroll into university, but they will drop out over time.<br><br>P4 I think this way is OK, meaning that these exams are given [to students], Croatian, mathematics, English… you will get students that are willing to learn, who have knowledge. Because this is the kind of exams they are. They are at high levels, you have to have a high level of knowledge to solve them.
Interviews could be a good selection method	P1: In my opinion, there should be a kind of oral exam… like in, I don't know the exact name, [anonymized], they had all those oral exams where they would see

	what kind of person someone is. So that they don't allow a person to enroll who isn't made for it.<br><br>P1: I would put in this oral [exam] to see what affinities a person has, that this is one part of it. What their personality and interests are. (…) I would make a small psychological interview. To see if this is a stable person.<br><br>P4: When looking at the, let's say, psychological side of things… it would be good to include some form of interviews. Although this would not be a guarantee that all [good] people would be selected. (…) In any case, it would be good to find people, right at the start, who, let's say, have a larger probability of… developing some sort of disorder in the future.
**Predictors of academic success in medical school**	
Perseverance	I: What is, in your opinion, the main predictor that students have a good GPA?<br><br>P1: Perseverance.<br><br>P2: I think it's perseverance and a kind of motivation. Someone has a good GPA because they want to prove to themselves that they can. Someone needs a GPA above 4 or 5 for a scholarship. So I think it's more individual.<br><br>P3: I don't think it's some kind of logical intelligence, but purely how long you can sit with a textbook.<br><br>P4: Perseverance. Work.

Abbreviations: SGE=State Graduation Exam, GPA=grade point average.
